# Supplementary material for: A cerebellar substrate for cognition evolved multiple times independently in mammals
Source: eLife. 2018 May 29;7:e35696. doi: 10.7554/eLife.35696 (PMC6003771; doi:10.7554/eLife.35696)
Supplement: Figure 2—source data 1. [file elife-35696-fig2-data1.docx]

| Species | Brain | Medial | Cerebellum | Source |
| --- | --- | --- | --- | --- |
| Loxodonta africana | 5145.00 | 85.23 | 946.36 | Maseko et al. 2012 |
| Procolobus badius | 78.70 | 2.69 | 8.43 | Smaers et al. 2011 |
| Nasalis larvatus | 64.25 | 2.63 | 7.65 | Smaers et al. 2011 |
| Macaca mulatta | 90.56 | 2.63 | 8.09 | MacLeod et al. 2003 |
| Lophocebus albigena | 112.00 | 4.00 | 11.70 | MacLeod et al. 2003 |
| Papio anubis | 191.00 | 5.19 | 17.71 | Smaers et al. 2011 |
| Papio cynocephalus | 144.80 | 3.78 | 13.83 | MacLeod et al. 2003 |
| Mandrillus sphinx | 155.90 | 3.51 | 16.23 | Maseko et al. 2012 |
| Cercocebus torquatus | 98.85 | 2.30 | 8.65 | MacLeod et al. 2003 |
| Erythrocebus patas | 89.00 | 2.70 | 7.90 | MacLeod et al. 2003 |
| Miopithecus talapoin | 41.10 | 1.25 | 3.70 | Smaers et al. 2011 |
| Cercopithecus ascanius | 61.50 | 1.73 | 4.94 | Smaers et al. 2011 |
| Cercopithecus mitis | 75.00 | 2.00 | 5.84 | Smaers et al. 2011 |
| Hylobates lar | 91.37 | 2.16 | 12.19 | MacLeod et al. 2003 |
| Pongo pygmaeus | 372.31 | 4.77 | 45.01 | MacLeod et al. 2003 |
| Gorilla gorilla | 401.28 | 6.30 | 59.38 | MacLeod et al. 2003 |
| Pan paniscus | 322.65 | 6.48 | 47.30 | MacLeod et al. 2003 |
| Pan troglodytes | 350.93 | 5.09 | 46.28 | MacLeod et al. 2003 |
| Homo sapiens | 1265.11 | 11.75 | 139.16 | MacLeod et al. 2003 |
| Aotus trivirgatus | 19.13 | 0.90 | 1.75 | MacLeod et al. 2003 |
| Saimiri sciureus | 22.02 | 0.90 | 2.00 | MacLeod et al. 2003 |
| Cebus albifrons | 76.50 | 2.35 | 7.05 | MacLeod et al. 2003 |
| Cebus apella | 66.45 | 1.83 | 6.45 | MacLeod et al. 2003 |
| Ateles geoffroyi | 106.40 | 2.80 | 11.16 | Smaers et al. 2011 |
| Ateles paniscus | 90.70 | 3.03 | 10.37 | MacLeod et al. 2003 |
| Alouatta seniculus | 37.40 | 1.45 | 4.15 | MacLeod et al. 2003 |
| Pithecia pithecia | 34.00 | 1.32 | 3.58 | Smaers et al. 2011 |
| Nycticebus coucang | 13.35 | 0.73 | 1.42 | Maseko et al. 2012 |
| Eulemur mongoz | 21.80 | 0.70 | 1.51 | Maseko et al. 2012 |
| Lama guanicoe | 200.30 | 9.39 | 22.46 | Maseko et al. 2012 |
| Sus scrofa | 95.30 | 4.47 | 9.28 | Maseko et al. 2012 |
| Tursiops truncatus | 1500.00 | 22.42 | 258.75 | Maseko et al. 2012 |
| Phocoena phocoena | 494.50 | 5.81 | 70.46 | Maseko et al. 2012 |
| Odocoileus virginianus | 160.00 | 10.96 | 18.75 | Maseko et al. 2012 |
| Bos taurus indicus | 520.50 | 16.26 | 33.71 | Maseko et al. 2012 |
| Ursus maritimus | 458.60 | 13.87 | 51.95 | Maseko et al. 2012 |
| Phoca vitulina | 275.00 | 3.93 | 24.01 | Maseko et al. 2012 |
| Zalophus californianus | 379.13 | 6.68 | 58.19 | Maseko et al. 2012 |
| Ailurus fulgens | 41.68 | 1.68 | 4.88 | Maseko et al. 2012 |
| Nasua narica | 37.00 | 1.10 | 3.49 | Maseko et al. 2012 |
| Bassariscus astutus | 20.70 | 0.92 | 2.00 | Maseko et al. 2012 |
| Mustela putorius | 8.30 | 0.54 | 1.12 | Maseko et al. 2012 |
| Mustela erminea | 4.00 | 0.18 | 0.40 | Maseko et al. 2012 |
| Neovison vison | 8.50 | 0.51 | 1.17 | Maseko et al. 2012 |
| Galictis vittata | 24.30 | 0.74 | 1.77 | Maseko et al. 2012 |
| Vulpes vulpes | 43.50 | 1.61 | 4.59 | Maseko et al. 2012 |
| Panthera leo | 258.00 | 9.87 | 26.54 | Maseko et al. 2012 |
| Felis silvestris | 36.90 | 1.88 | 4.19 | Maseko et al. 2012 |
| Crocuta crocuta | 162.50 | 6.06 | 15.84 | Maseko et al. 2012 |
| Cynictis penicillata | 14.48 | 0.73 | 1.34 | Maseko et al. 2012 |
