# Supplementary material for: A cerebellar substrate for cognition evolved multiple times independently in mammals
Source: eLife. 2018 May 29;7:e35696. doi: 10.7554/eLife.35696 (PMC6003771; doi:10.7554/eLife.35696)
Supplement: Supplementary file 1. [file elife-35696-supp1.docx]

Table 1 – supplement 1

|  | pANCOVA | | | | |
| --- | --- | --- | --- | --- | --- |
| Group allocation | Grouping | *df* | *F* | *P* | |
| High = Musteline carnivorans | Among groups | 2,46 | 18.076 | <0.001 | *** |
| Medium = Others | High v Medium \| Low | 1,46 | 13.230 | <0.001 | *** |
| Low = Cercopithecine primates and zebu | Medium v Low \| High | 1,46 | 19.033 | <0.001 | *** |
| High = Others | Among groups | 2,46 | 13.549 | <0.001 | *** |
| Medium = Cercopithecine primates | High v Medium \| Low | 1,46 | 13.614 | <0.001 | *** |
| Low = Zebu | Medium v Low \| High | 1,46 | 6.703 | 0.013 | * |
|  |  |  |  |  |  |
